# Soluble PD-L1 as a novel biomarker predicts poor outcomes and disease progression in *de novo* myelodysplastic syndromes

**DOI:** 10.1186/s40364-024-00665-y

**Published:** 2024-10-08

**Authors:** Xingcheng Yang, Lijun Jiang, Xiaoying Zhang, Juan Peng, Hu Qian, Lifang Huang, Shaolong He, Zhiqiong Wang, Liting Chen, Yicheng Zhang, Ling Ma, Yuan Chen, Jia Wei

**Affiliations:** 1 Department of Hematology, Tongji Hospital, Tongji Medical College, Huazhong University of Science and Technology, Wuhan, 430030 Hubei China; 2 Department of Hematology, Shanxi Bethune Hospital, Shanxi Academy of Medical Sciences, Tongji Shanxi Hospital, Third Hospital of Shanxi Medical University, Taiyuan, 030032 Shanxi China; 3 Department of Geriatrics, Tongji Hospital, Tongji Medical College, Huazhong University of Science and Technology, Wuhan, 430030 Hubei China; 4 Sino-German Joint Oncological Research Laboratory, Shanxi Bethune Hospital, Shanxi Academy of Medical Sciences, Taiyuan, 030032 Shanxi China; 5 Department of Geriatrics, Tongji Shanxi Hospital, Shanxi Bethune Hospital, Shanxi Academy of Medical Sciences, Third Hospital of Shanxi Medical University, Taiyuan, 030032 Shanxi China; 6 Department of Clinical Laboratory, Union Hospital, Tongji Medical College, Huazhong University of Science and Technology, Wuhan, 430022 China; 7 Key Laboratory of Organ Transplantation, NHC Key Laboratory of Organ Transplantation, Key Laboratory of Organ Transplantation, Ministry of Education, Chinese Academy of Medical Sciences, Wuhan, China; 8 Immunotherapy Research Center for Hematologic Diseases of Hubei Province, Wuhan, 430030 Hubei China

**Keywords:** Myelodysplastic syndromes, PD-1/PD-L1 signaling pathway, Soluble immune checkpoints, Cytokines, Hypomethylating agents

## Abstract

The role of the compromised immune microenvironment, including immune checkpoints, in myelodysplastic syndromes (MDS) has been identified as critical This study aimed to investigate the expression patterns of immune checkpoints, particularly soluble PD-1/PD-L1 (sPD-1/sPD-L1) as well as PD-1 on effector T cell subsets, and assess their prognostic value and potential regulatory roles in MDS. 161 MDS patients were enrolled, including 129 patients were primarily diagnosed with *de novo* MDS, together with 59 MDS patients who underwent hypomethylating agents (HMAs) therapy. Plasma sPD-L1 level was elevated in newly diagnosed MDS patients, which was also found to be associated with MDS disease progression that further increase in higher IPSS-R score group. Patients with increased sPD-L1 expression at diagnosis exhibited notably poorer overall survival, and multivariate Cox analysis indicated that elevated sPD-L1 was an independent risk factor. Furthermore, the levels of multiple cytokines and membrane-bound PD-1 on T cells were found to correlate with sPD-1/sPD-L1 levels in plasma. Importantly, we also found sPD-L1 levels significantly increased in MDS patients who showed progression of disease following HMAs therapy. In conclusion, we found elevated plasma sPD-L1 levels in MDS patients are associated with disease progression and poorer overall survival. This study showed that sPD-L1 is a potential biomarker for prognosis and a target for immunotherapy in MDS.

**Supplementary Information:**

The online version contains supplementary material available at 10.1186/s40364-024-00665-y.

**To the editor**.

Myelodysplastic syndromes (MDS) are characterized by ineffective hematopoiesis, genetic abnormalities, clonal hematopoiesis, and a high risk of progression to acute myeloid leukemia (AML) [1]. Hypomethylating agents (HMAs) like decitabine (DAC) and azacitidine (AZA) are primary treatments for advanced MDS patient’s ineligible for allogeneic hematopoietic stem cell transplantation (HSCT), but only about half respond, and responses are generally transient [2]. Patients unresponsive to HMAs have poor survival, with a median overall survival (OS) of 4.3 to 5.6 months [3]. The PD-1/PD-L1 pathway plays a critical role in MDS [4, 5]. Clinical trials are exploring PD-1/PD-L1 inhibitors alone or with HMAs, but efficacy remains limited, necessitating further investigation into the mechanisms. Previous research suggests these soluble immune checkpoints (sICs) could predict prognosis and foster resistance to ICIs [6–10], However, in the context of MDS, the potential dysregulation and prognostic significance of sICs remain uncertain.

In this study, we evaluate the roles of sPD-1/sPD-L1 and their prognostic relevance in MDS. We enrolled a total of 161 MDS patients (129 patients were primarily diagnosed with *de novo* MDS, together with 59 MDS patients who underwent HMAs only, from July 2020 to October 2023 (Supplementary Fig. S1). MDS patients were categorized by IPSS-R as lower-risk (very low risk/low risk) or higher-risk (intermediate risk/high risk/very high risk) (Supplementary Tables S1-S2) [11].

We found sPD-L1 levels were significantly elevated in the 129 newly diagnosed MDS patients (median 75.26 pg/ml, range 25.00-252.6 pg/ml) while sPD-1 levels (median 149.7 pg/ml, range 32.04–502.3 pg/ml) were lower compared to healthy controls (Fig. 1A-B). Other soluble immune checkpoints, including sCTLA-4, sGITR, sLAG-3, s4-1BB, and sTIM-3, also exhibited lower levels (Supplementary Fig. S2). Additionally, sPD-L1 levels were significantly higher in the higher-risk groups of newly diagnosed MDS patients, however, no significant differences were observed in other soluble immune checkpoints (Fig. 1C, Supplementary Fig. S3). Through paired analysis, we found no significant difference in sPD-L1 levels between blood plasma and bone marrow samples. (Supplementary Fig. S4).

**Fig. 1:**
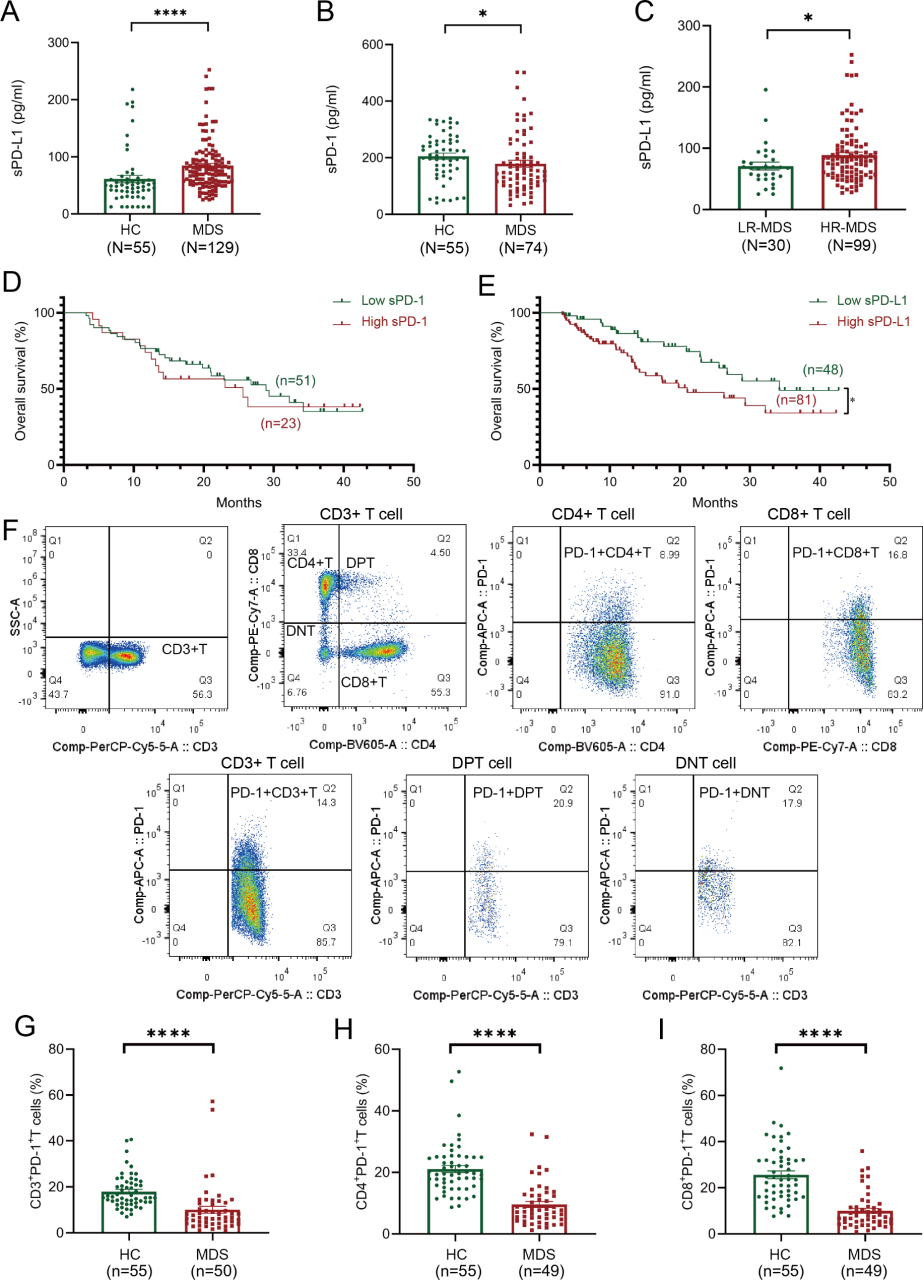
Plasma sPD-1/sPD-L1 levels between healthy donors and newly diagnosed MDS patients. (**A**) Comparison of sPD-L1 levels in plasma from MDS and HC. (**B**) Comparison of sPD-1 levels in plasma from MDS and HC. (**C**) Comparison of sPD-L1 levels in plasma between LR-MDS and HR-MDS. (**D**) OS according to low (< 187.5 pg/mL) and high (> 187.5 pg/mL) pretreatment plasma sPD-1 levels. (**E**) OS according to low (< 66.38 pg/mL) and high (> 66.38 pg/mL) pretreatment plasma sPD-L1 levels. (**F**) The gating strategy used for T lymphocyte subsets, including CD3^+^, CD4^+^, CD8^+^, DPT, DNT and PD-1^+^. (**G**) Comparison of CD3^+^ PD-1^+^ T cells percentage in PB between MDS and HC. (**H**) Comparison of CD4^+^ PD-1^+^ T cells percentage in PB between MDS and HC. (**I**) Comparison of CD8^+^ PD-1^+^ T cells percentage in PB between MDS and HC. LR, lower-risk; HR, higher risk; DPT, double-positive T cells; DNT, double-negative T cells

The AUC for sPD-1 was 0.6252 [95% CI 0.5250–0.7253, *P* < 0.05], for sPD-L1 was 0.7284 [95% CI 0.6412–0.8156, *P* < 0.0001]. The optimal cut-off values for sPD-1 were 187.5 pg/mL and for sPD-L1 were 66.38 pg/mL. Patients with elevated sPD-L1 showed shorter OS (*P* = 0.0314) (Fig. 1D-E). Through univariate Cox regression analysis, we found high sPD-L1 expression (*P* = 0.034, HR = 1.925, 95% CI: 1.049–3.530), elevated IPSS-R scores, transfusion dependence and age were associated with poorer OS. Upon adjusting for pertinent indicators, we determined that high sPD-L1 expression (*P* = 0.008, HR = 4.172, 95% CI: 1.265–13.755), elevated IPSS-R scores, age and transfusion dependence were independently risk factors for poor OS in newly diagnosed patients (Supplementary Tables S3).

Flow cytometry was employed to analyze the proportions of PD-1^+^ cells within T cell subsets in the peripheral blood of newly diagnosed MDS patients (Fig. 1F). We found PD-1^+^ T cell subsets were diminished in MDS (Fig. 1G-I). Nevertheless, no significant distinction between higher and lower-risk MDS groups was observed (Supplementary Fig. S5). Positive correlations between sPD-1 levels and both the proportion and absolute count of PD-1^+^ CD4^+^ T cells, as well as the absolute count of PD-1^+^ double-positive T cells (DPT) were noticed. (Fig. 2A-D).

**Fig. 2:**
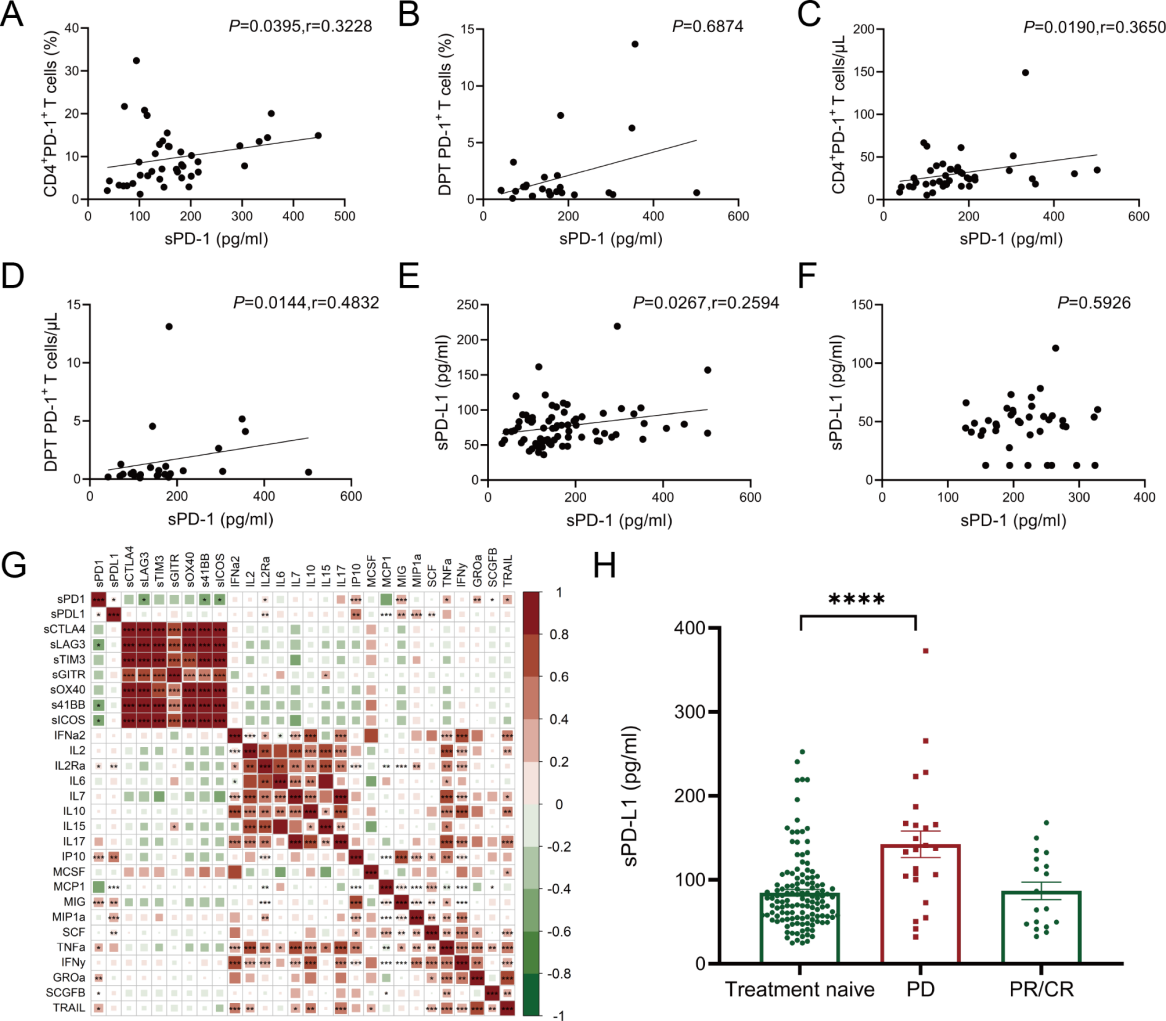
Correlation and Analysis of sPD-1 and sPD-L1 Levels in MDS Patients. (**A**) percentages of CD4^+^ PD-1^+^ T cells and serum levels of sPD-1. (**B**) percentages of PD-1^+^ DPT cells and serum levels of sPD-1. (**C**) absolute count of CD4^+^ PD-1^+^ T cells and serum levels of sPD-1. (**D**) absolute count of PD-1^+^ DPT cells and serum levels of sPD-1. (**E**) plasma sPD-1 and sPD-L1 in MDS patients. (**F**) Correlation between the levels of plasma sPD-1 and sPD-L1 in HC. (**G**) Spearman rank correlation analysis was used to analyze the correlation between various indicators, and the correlation coefficients and P values were calculated. Red indicates a positive correlation between two indicators, and green indicates a negative correlation between two indicators, deeper the color, the larger the correlation coefficient. (**H**) Plasma sPD-L1 levels in treatment-naive MDS patients, PD patients, and CR/PR patients

We explored the relationship between sPD-1 and sPD-L1 levels and identified a positive correlation in newly diagnosed MDS patients, but was absent in healthy individuals, suggesting its relevance to the MDS disease state (Fig. 2E-F). Subsequently, we explored the connection between plasma cytokines, chemokines and soluble immune checkpoints, we found that sPD-1 levels exhibited positive associations with GROα, IP-10, MIG, TRAIL, TNFα, IL-2Rα, and sPD-L1, while displaying negative correlations with sLAG-3, s4-1BB, and sICOS. Additionally, sPD-L1 levels exhibited positive correlations with SCF, MIG, MCP-1, IL-2Rα, IP-10, and MIP1α (Fig. 2G).

To further investigate the impact of HMAs on sPD-1/sPD-L1 levels, we analyzed a cohort of 59 MDS patients who had received at least two cycles of HMA therapy without any other chemotherapy regimen in the past six months. Among the 59 MDS patients, 12 achieved CR or mCR, 6 achieved PR, 24 experienced PD, and 17 maintained SD. We observed an increase in sPD-L1 levels in MDS patients who showed PD following HMA treatment (Fig. 2H).

In conclusion, our data demonstrate the prognostic significance of sPD-L1 in MDS. Elevated plasma sPD-L1 levels were associated with higher IPSS-R scores, poorer OS, and an increased likelihood of disease progression following demethylation therapy, underscoring sPD-L1 as a valuable prognostic biomarker. While our study focused on sPD-L1, mPD-L1 also plays critical roles in the tumor microenvironment and may have potential clinical implications. Further investigation into the relationship between sPD-L1 and mPD-L1 could offer a more comprehensive understanding of their roles in MDS pathogenesis. Incorporating sPD-L1 levels into clinical practice could improve risk stratification and guide treatment planning for MDS patients. Additionally, future studies should explore combinations of HMAs with novel agents and immunotherapy in the treatment of MDS [12].

## Electronic supplementary material

Below is the link to the electronic supplementary material.

Supplementary Material 1

## Data Availability

The datasets analyzed during the current study are available from the corresponding author on reasonable request.

## Abbreviations

AML: Acute myeloid leukemia
AUC: Area under the curve
AZA: Azacitidine
DAC: Decitabine
DPT: Double-positive T cells
HMAs: Hypomethylating agents
HSCT: Hematopoietic stem cell transplantation
MDS: Myelodysplastic syndromes
OS: Overall survival
PD-1: Programmed cell death-1
PD-L1: Programmed cell death ligand-1
ROC: Receiver operating characteristic
sICs: Soluble immune checkpoints
