# Supplementary material for: Soluble PD-L1 as a novel biomarker predicts poor outcomes and disease progression in de novo myelodysplastic syndromes
Source: Biomark Res. 2024 Oct 8;12:115. doi: 10.1186/s40364-024-00665-y (PMC11463091; doi:10.1186/s40364-024-00665-y)
Supplement: Supplementary file 1 — Supplementary Material 1 [file 40364_2024_665_MOESM1_ESM.docx]

**Supplementary Files**

Soluble PD-L1 as a novel biomarker predicts poor outcomes and disease progression in de novo myelodysplastic syndromes

Table of Contents

[Supplementary Materials and Methods 1](#_Toc178187638)

[Supplementary Table S1. Patient baseline characteristics. 5](#_Toc178187639)

[Supplementary Table S2. Correlations of sPD-1/sPD-L1 between clinical characteristics in newly diagnosed MDS patients. 6](#_Toc178187640)

[Supplementary Table S3. Multivariate Cox regression analysis of PD-1/PD-L1 levels and OS. 7](#_Toc178187641)

[Supplementary Table S4. Sensitivity and assay range of ELISA kit and multiplex cytokine screening panel. 8](#_Toc178187642)

[Supplementary Figure S1. Flow diagram of patients screening 9](#_Toc178187643)

[Supplementary Figure S2. Plasma sICs levels between newly diagnosed MDS patients and healthy controls. 10](#_Toc178187644)

[Supplementary Figure S3. Plasma sICs levels between lower-risk group and higher-risk group of MDS patients. 11](#_Toc178187645)

[Supplementary Figure S4. Plasma and bone marrow sICs levels of newly diagnosed MDS patients. 12](#_Toc178187646)

[Supplementary Figure S5. PD-1 expression on T cell subpopulations between lower-risk group and higher-risk group of MDS patients. 13](#_Toc178187647)

# Supplementary Materials and Methods

**Patient enrollment and inclusion** **criteria**

From July 2020 to October 2023, this study enrolled a total of 161 MDS patients (Supplementary Fig. S1). One hundred and twenty-nine patients were primarily diagnosed with *de novo* MDS, together with 59 MDS patients who underwent HMAs only and had no prior exposure to chemotherapy in the past 6 months were also enrolled in our study. The inclusion criteria for patients were: (1) a confirmed pathological diagnosis of MDS based on 2016/2022 WHO classification; (2) availability of complete clinical information and survival data for at least 3 months; and (3) absence of autoimmune diseases or other types of tumors. Exclusion criteria included: (1) evidence of active infection, confirmed by either culture-based or non-culture-based methods, along with relevant clinical signs and symptoms; and (2) an ECOG performance score of ≥ 3. MDS patients were categorized by IPSS-R as lower-risk (very low risk/low risk) or higher-risk (intermediate risk/high risk/very high risk). The response to HMAs was assessed in accordance with the International Working Group (IWG) 2006 criteria [^1^](#_ENREF_1). Follow-up was conducted until April 16th, 2024. The median follow-up was 13.4 months (range 3.2-42.7 months). This study adhered to the principles of the Declaration of Helsinki and received approval from the Medical Ethics Committee of Tongji Hospital, Tongji Medical College, Huazhong University of Science and Technology.

**Measurement of sICs and cytokines**

Plasma and bone marrow specimens were collected into EDTA tubes and then centrifuged at 4°C (1500g for 5 minutes) within 4 hours and isolated cell-free plasma samples were stored at – 80 °C. Soluble immune checkpoints (sICs), including sPD-L1, sPD-1, s4-1BB, sGITR, sLAG-3, sTIM-3, sOX40, sICOS, and sCTLA-4 were evaluated using commercial ELISA kits (sPD-L1 and sPD-1: R&D Systems, Minneapolis, USA; s4-1BB, sGITR, sLAG-3, sTIM-3, sOX40, sICOS, sCTLA-4: BIOYE, Shanghai, China). Cytokines and chemokines (IFN-α, IL-2Rα, IL-15, IL-7, IL-17, IP10, MCSF, MCP1, IL-10, MIG, MIP1α, SCF, TNF-α, IFN-γ, IL-2, GRO-α, IL-6, SCGF-β, TRAIL) were assessed using Luminex analysis with commercial assays (Bio-Rad, CA, USA) (Supplementary Tables S4).

**Flow cytometric analysis**

Peripheral blood (PB) T cell subpopulations were analyzed using flow cytometry. The examination of circulating CD3^+^ PD-1^+^ T, CD8^+^ PD-1^+^ T, CD4^+^ PD-1^+^ T, PD-1^+^ double-positive T cells (DPT) and PD-1^+^ double-negative T cells (DNT) was carried out through the Fluorescence Activated Cell Sorting (FACS). Data processing was performed using the FACSCanto II flow cytometer (BD Biosciences, New Jersey, USA) and FACSDiva Software.

**Statistical analysis**

Overall survival (OS) was defined as the time from diagnosis to either death or the last follow-up. The Kaplan–Meier method was used to estimate OS, with group comparisons made using the log-rank test. The cut-off date for survival analysis was April 16th, 2024. Receiver Operating Characteristic (ROC) curves were utilized to determine marker sensitivity and specificity, reporting the area under the curve (AUC). To identify independent prognostic factors, we utilized univariate and multivariate Cox proportional hazards regression models, with the latter incorporating covariates that were statistically significant in the univariate analysis (P< 0.05). Differences between groups were analyzed using the Mann-Whitney U test for continuous variables and Fisher’s exact test for categorical variables. All statistical analyses were conducted using R software (version 4.2.3) and GraphPad Prism (version 8.0.2).

**References**

1. Cheson BD, Greenberg PL, Bennett JM, et al. Clinical application and proposal for modification of the International Working Group (IWG) response criteria in myelodysplasia. Blood. 2006;108(2):419-425.

**Supplementary Tables**

# Supplementary Table S1. Patient baseline characteristics.

| **MDS patients** | Treatment-naive^#^ |
| --- | --- |
| No | 129 |
| Mean age (range) | 60 (18-89) |
| Sex | 44 Female/85 Male |
| Median ANC (range), ×10^9^ /L | 0.95 (0.04-47.14) |
| Median hemoglobin (range), g/L | 74.0 (36.0-135.0) |
| Median platelets (range), ×10^9^ /L | 48.0 (5.0-357.0) |
| Median BM blast % (range) | 4.0 (0-18.0) |
| **Classification of WHO 2022** |  |
| MDS-5q | 5 |
| MDS-SF3B1 | 5 |
| MDS-biTP53 | 3 |
| MDS-LB | 37 |
| MDS-h | 4 |
| MDS-IB1 | 34 |
| MDS-IB2 | 39 |
| MDS-f | 2 |
| **IPSS-R** |  |
| Very low | 2 |
| Low | 28 |
| Intermediate | 37 |
| High | 36 |
| Very high | 26 |
| **Cytogenetic risk^*^** |  |
| Very good | 2 |
| Good | 95 |
| Intermediate | 14 |
| Poor | 6 |
| Very poor | 12 |

Treatment-naive^#^: patients who have received no therapy or only received supportive treatments such as blood transfusions; iron chelation therapy; stimulating factors including EPO, G-CSF, etc.; Cytogenetic risk*: determined according to the IPSS-R scoring system.

# Supplementary Table S2. Correlations of sPD-1/sPD-L1 between clinical characteristics in newly diagnosed MDS patients.

| **Characteristic** | sPD-1^*^ |  |  | sPD-L1^**^ |  |  |
| --- | --- | --- | --- | --- | --- | --- |
|  | Median, range | P | n | Median, range | P | n |
| **Gender** |  |  |  |  |  |  |
| Male | 156.5 (53.7-502.3) | 0.171 | 49 | 79.69 (25.0-219.6) | 0.1068 | 85 |
| Female | 139.9 (32.0-501.8) |  | 25 | 69.64 (25.0-252.6) |  | 44 |
| **Age** |  |  |  |  |  |  |
| ＜65 years | 146.4 (32.0-501.8) | 0.265 | 45 | 69.65 (25.0-252.6) | 0.0606 | 75 |
| ≥ 65 years | 159.0 (41.8-502.3) |  | 29 | 84.32 (28.1-219.6) |  | 54 |
| **Transfusion dependency** ^#^ |  |  |  |  |  |  |
| No | 151.0 (32.0-448.4) | 0.750 | 34 | 74.9 (25.0-252.6) | 0.650 | 64 |
| Yes | 149.7 (37.7-502.3) |  | 40 | 75.3 (25.0-240.8) |  | 65 |
| **Hemoglobin, g/L** |  |  |  |  |  |  |
| ＜80 g/L | 154.4 (32.0-502.3) | 0.384 | 45 | 78.2 (32.0-252.6) | 0.221 | 77 |
| ≥ 80 g/L | 144.0 (37.7-448.4) |  | 29 | 71.3 (25.0-219.6) |  | 52 |
| **Platelets, ×10^9^ /L** |  |  |  |  |  |  |
| ＜50 g/L | 148.0 (32.0-356.9) | 0.675 | 39 | 74.4 (25.0-219.6) | 0.488 | 63 |
| ≥50 g/L | 144.0 (37.7-502.3) |  | 35 | 76.5 (25.0-252.6) |  | 66 |
| **ANC, ×10^9^ /L** |  |  |  |  |  |  |
| ＜0.8 ×10^9^ /L | 151.4 (64.0-356.9) | 0.663 | 31 | 69.3 (27.0-161.9) | 0.167 | 54 |
| ≥0.8×10^9^ /L | 148.0 (32.0-502.3) |  | 43 | 78.2 (25.0-252.6) |  | 75 |
| **BM blast %** |  |  |  |  |  |  |
| ＜5% | 157.0 (37.7-448.4) | 0.854 | 41 | 78.2 (25.0-252.6) | 0.810 | 67 |
| ≥5% | 148.0 (32.0-502.3) |  | 33 | 74.1 (27.0-219.6) |  | 62 |
| **IPSS-R**^***^ |  |  |  |  |  |  |
| Lower-risk | 110.0 (37.7-353.7) | 0.061 | 15 | 67.7 (25.0-195.5) | **0.038** | 30 |
| Higher-risk | 154.4 (32.0-502.3) |  | 59 | 80.4 (27.0-252.6) |  | 99 |

Transfusion dependence^#^: Patients who have received at least 2 transfusions of blood products, including plasma, platelets, red blood cells, coagulation factors, etc., with an interval of at least 5 days within one month after enrollment; sPD-1^*^: pg/mL; sPD-L1^**^: pg/mL; IPSS-R^***^: lower-risk (very low risk/ low risk), higher-risk (intermediate risk/high risk/very high risk).

# Supplementary Table S3. Multivariate Cox regression analysis of PD-1/PD-L1 levels and OS.

| Characteristic | Univariate | | | Multivariate | | |
| --- | --- | --- | --- | --- | --- | --- |
|  | 95% CI | HR | *P* | 95% CI | HR | *P* |
| Age | **1.003-1.051** | **1.026** | **0.030** | **1.003-1.049** | **1.026** | **0.024** |
| Sex | 0.591-2.057 | 1.103 | 0.759 |  |  |  |
| IPSS-R＞3.5 | **1.251-4.662** | **2.416** | **0.009** | **1.281-4.996** | **2.530** | **0.008** |
| Transfusion dependence | **1.634-5.579** | **3.019** | **0.0004** | **1.626-5.664** | **3.035** | **0.0004** |
| High sPD-1^*^ | 0.581-2.209 | 1.133 | 0.714 |  |  |  |
| High sPD-L1^*^ | **1.049-3.530** | **1.925** | **0.034** | **1.256-4.476** | **2.371** | **0.008** |
| High CD3^+^PD-1^+^ T (%) ^*^ | 0.759-3.988 | 1.739 | 0.191 |  |  |  |
| High CD4^+^PD-1^+^ T (%) ^*^ | 0.0995-1.831 | 0.427 | 0.252 |  |  |  |
| High CD8^+^PD-1^+^ T (%) ^*^ | 0.522-3.806 | 1.410 | 0.498 |  |  |  |

High expression^*^: Expression levels above the optimal cutoff value according to ROC curves.

# Supplementary Table S4. Sensitivity and assay range of ELISA kit and multiplex cytokine screening panel.

| Critical commercial assays | Source | Assay/control range | Sensitivity |
| --- | --- | --- | --- |
| Human PD-1 Quantikine ELISA Kit | R&D Systems | 15.6–10,000 pg/mL | 3.27 pg/mL |
| Human/Cynomolgus Monkey PD-L1/B7-H1 Quantikine ELISA Kit | R&D Systems | 25.0 - 1,600 pg/mL | 4.52 pg/mL |
| Human s4-1BB ELISA Kit | BIOYE | 12.29 – 3000 pg/mL | 10 pg/mL |
| Human sGITR ELISA Kit | BIOYE | 13.7 – 10000 pg/mL | 13 pg/mL |
| Human sLAG-3 ELISA Kit | BIOYE | 49.15 – 12000 pg/mL | 45 pg/mL |
| Human sTIM-3 ELISA Kit | BIOYE | 10.24 – 2500 pg/mL | 10 pg/mL |
| Human sOX40 ELISA Kit | BIOYE | 3.28 – 800 pg/mL | 3.2 pg/mL |
| Human sICOS ELISA Kit | BIOYE | 1.2 – 300 pg/mL | 1.2 pg/mL |
| Human sCTLA-4 ELISA Kit | BIOYE | 12 – 500 pg/mL | 12 pg/mL |
| Bio-Plex Pro Human Cytokine Screening Panel, 48-plex | Bio-Rad |  |  |
| IFN-α | - | 90-210 pg/mL | - |
| IL-2Rα | - | 120-302 pg/mL | - |
| IL-15 | - | 999-2331 pg/mL | - |
| IL-7 | - | 153-357 pg/mL | - |
| IL-17 | - | 179-418 pg/mL | - |
| IP-10 | - | 93-216 pg/mL | - |
| M-CSF | - | 51-118 pg/mL | - |
| MCP-1 | - | 38-88 pg/mL | - |
| IL-10 | - | 104-243 pg/mL | - |
| MIG | - | 16.16-37.72 pg/mL | - |
| MIP-1α | - | 6-14 pg/mL | - |
| SCF | - | 181-423 pg/mL | - |
| TNF-α | - | 87.61-204.41 pg/mL | - |
| IFN-γ | - | 90-210 pg/mL | - |
| IL-2 | - | 148-345 pg/mL | - |
| GRO-α | - | 384-897pg/mL | - |
| IL-6 | - | 31-72 pg/mL | - |
| SCGF-β | - | 14270-33296 pg/mL | - |
| TRAIL | - | 46-107 pg/mL | - |

**Supplementary Figures**

# Supplementary Figure S1. Flow diagram of patients screening


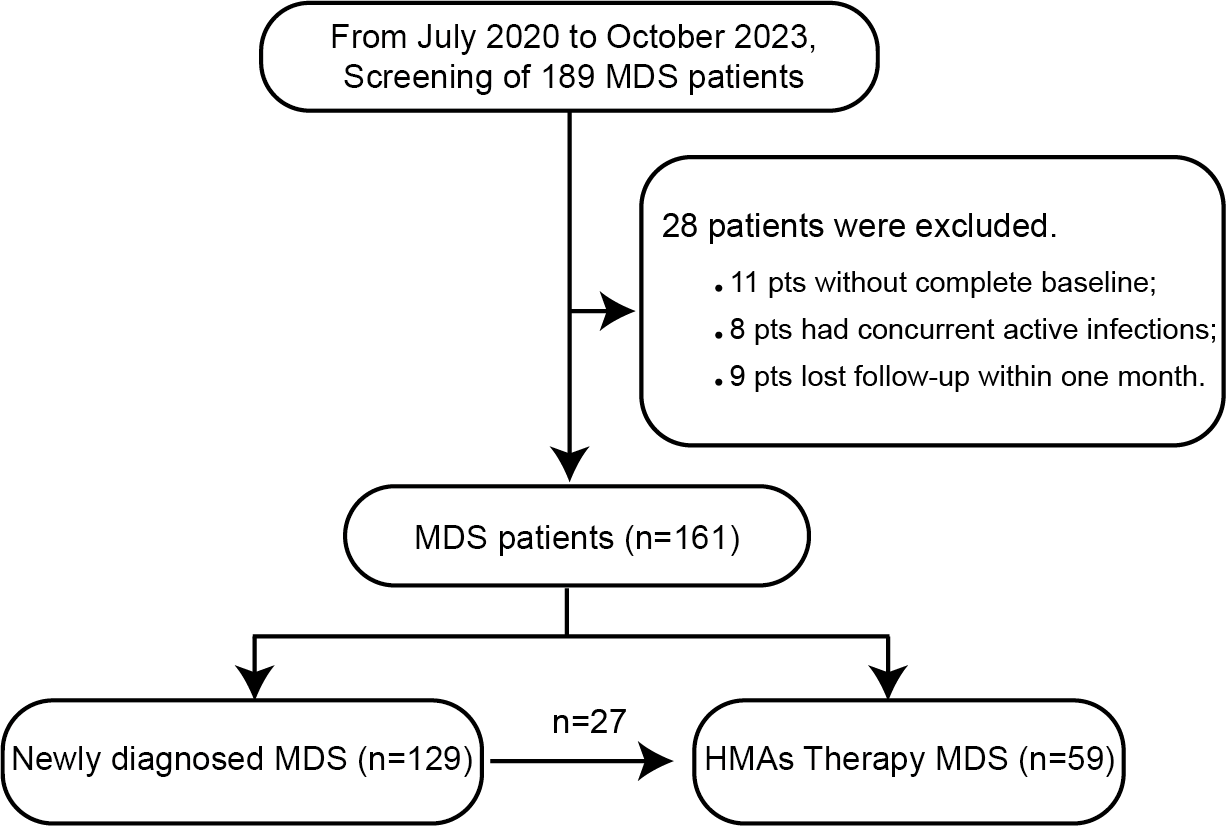


From July 2020 to October 2023, this study enrolled a total of 161 MDS patients. Of these, 129 were primarily diagnosed with de novo MDS, along with 59 MDS patients who underwent HMAs only and had no prior exposure to chemotherapy in the past 6 months. Additionally, 27 patients had samples available from both their initial and HMA treatment cohorts. The inclusion criteria for patients were: (1) a confirmed pathological diagnosis of MDS based on 2016/2022 WHO classification; (2) availability of complete clinical information and survival data for at least 3 months; and (3) absence of autoimmune diseases or other types of tumors. Exclusion criteria included: (1) evidence of active infection, confirmed by either culture-based or non-culture-based methods, along with relevant clinical signs and symptoms; and (2) an ECOG performance score of ≥ 3.

# Supplementary Figure S2. Plasma sICs levels between newly diagnosed MDS patients and healthy controls.


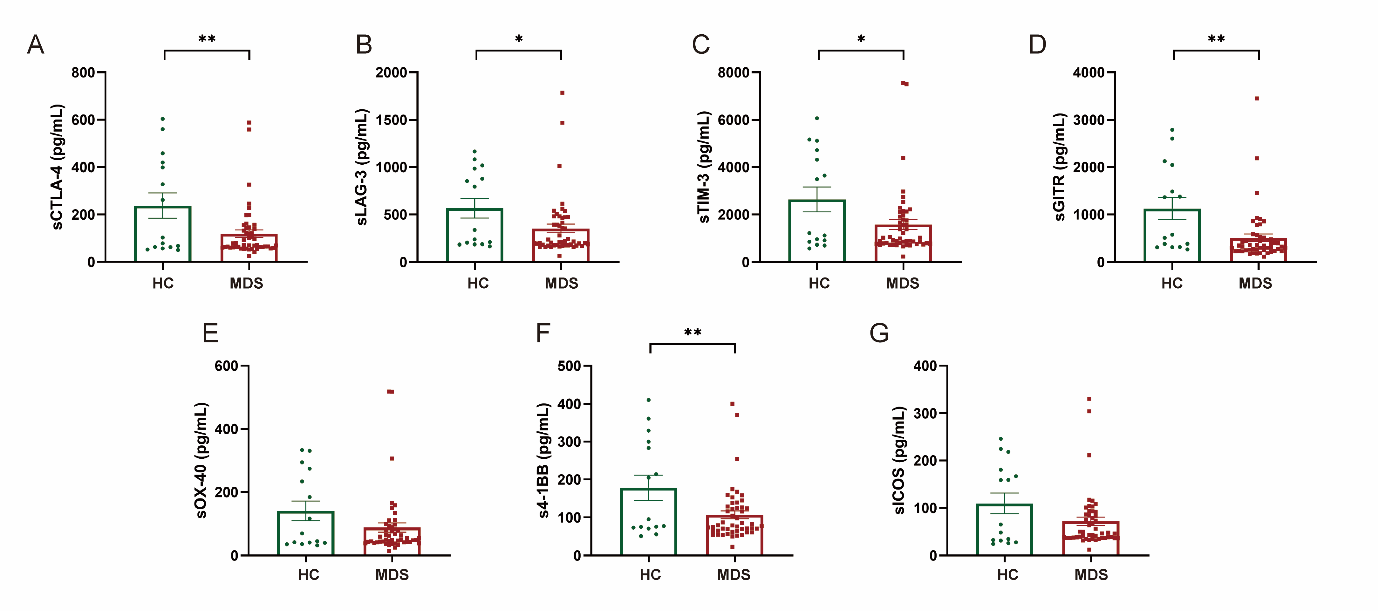


Comparison of plasma (A) sCTLA-4, (B) sLAG-3, (C) sTIM-3, (D) sGITR, (E) sOX-40, (F) s4-1BB, (G) sICOS between newly diagnosed MDS patients and healthy controls.

# Supplementary Figure S3. Plasma sICs levels between lower-risk group and higher-risk group of MDS patients.


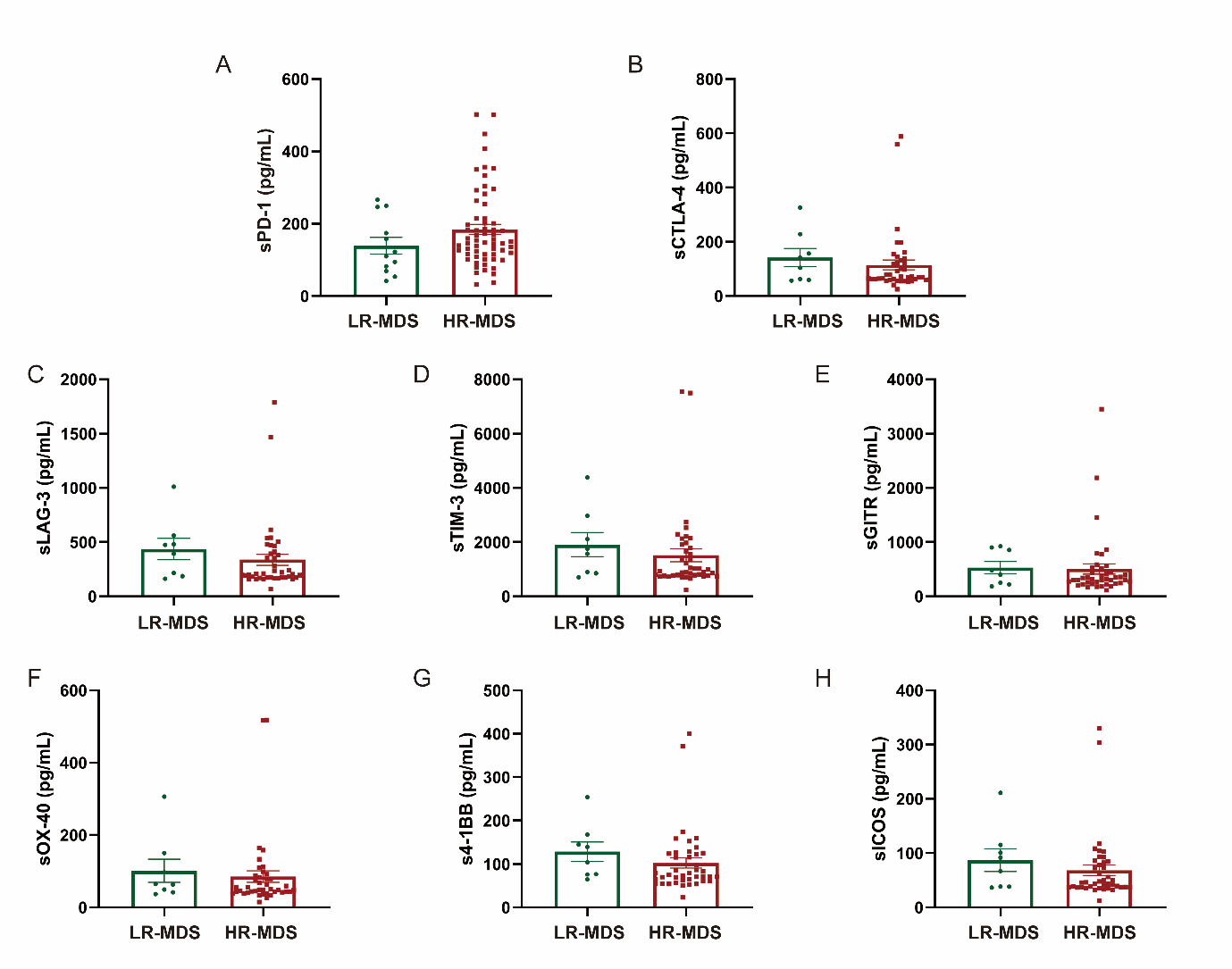


Comparison of plasma (A) sPD-1, (B) sCTLA-4, (C) sLAG-3, (D) sTIM-3, (E) sGITR, (F) sOX-40, (G) s4-1BB, (H) sICOS between lower-risk group and higher-risk group of newly diagnosed MDS patients.

# Supplementary Figure S4. Plasma and bone marrow sICs levels of newly diagnosed MDS patients.


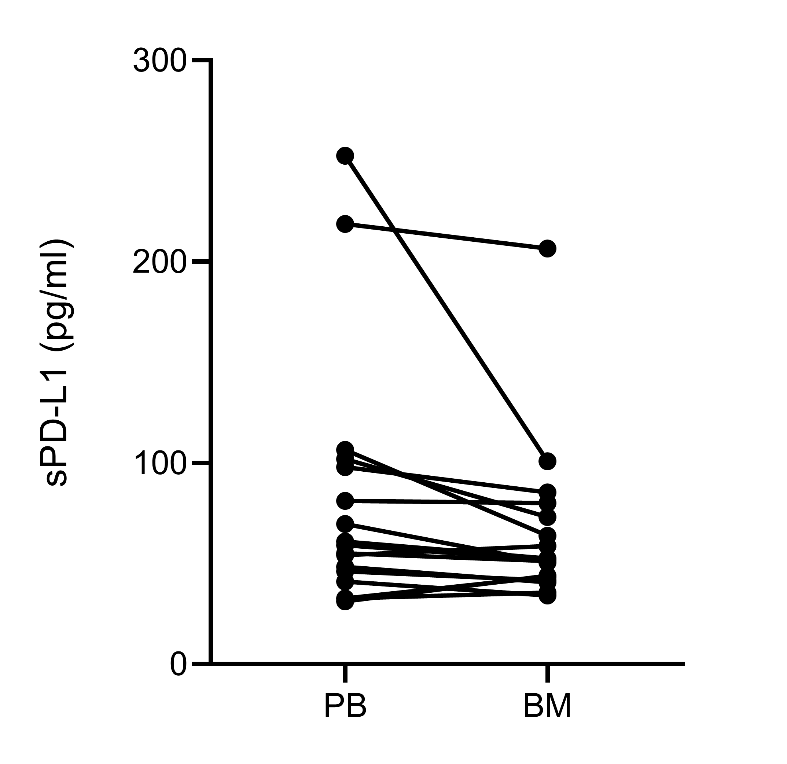


Comparison of plasma and bone marrow sICs levels of newly diagnosed MDS patients.

# Supplementary Figure S5. PD-1 expression on T cell subpopulations between lower-risk group and higher-risk group of MDS patients.


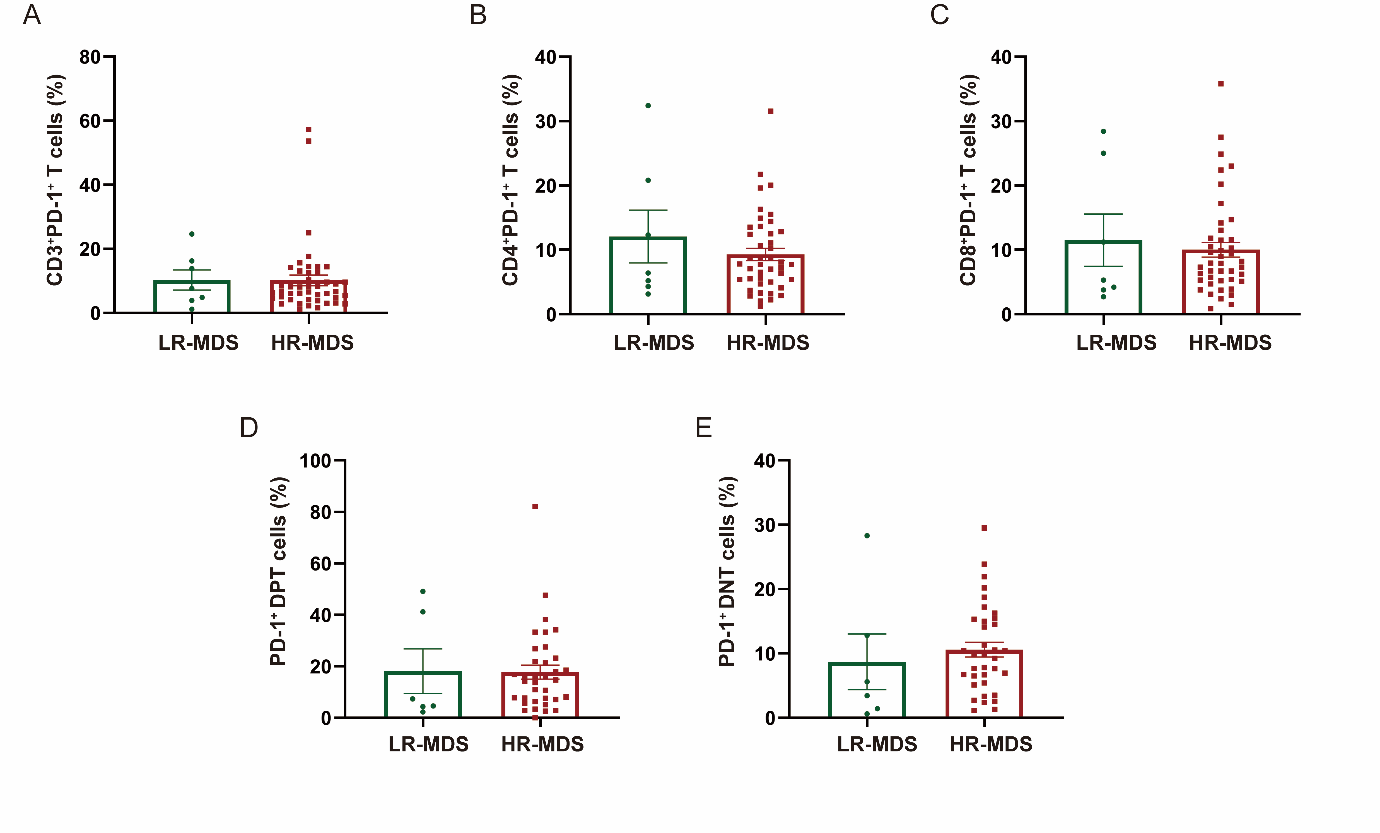


Comparison of (A) CD3^+^ PD-1^+^ T cells (B) CD4^+^ PD-1^+^ T cells, (C) CD8^+^ PD-1^+^ T cells, (D) DPT PD-1^+^ cells, (E) DNT PD-1^+^ cells.
